# Supplementary figures and images for: CoMA – an intuitive and user-friendly pipeline for amplicon-sequencing data analysis
Source: PLoS One. 2020 Dec 2;15(12):e0243241. doi: 10.1371/journal.pone.0243241 (PMC7710066; doi:10.1371/journal.pone.0243241)

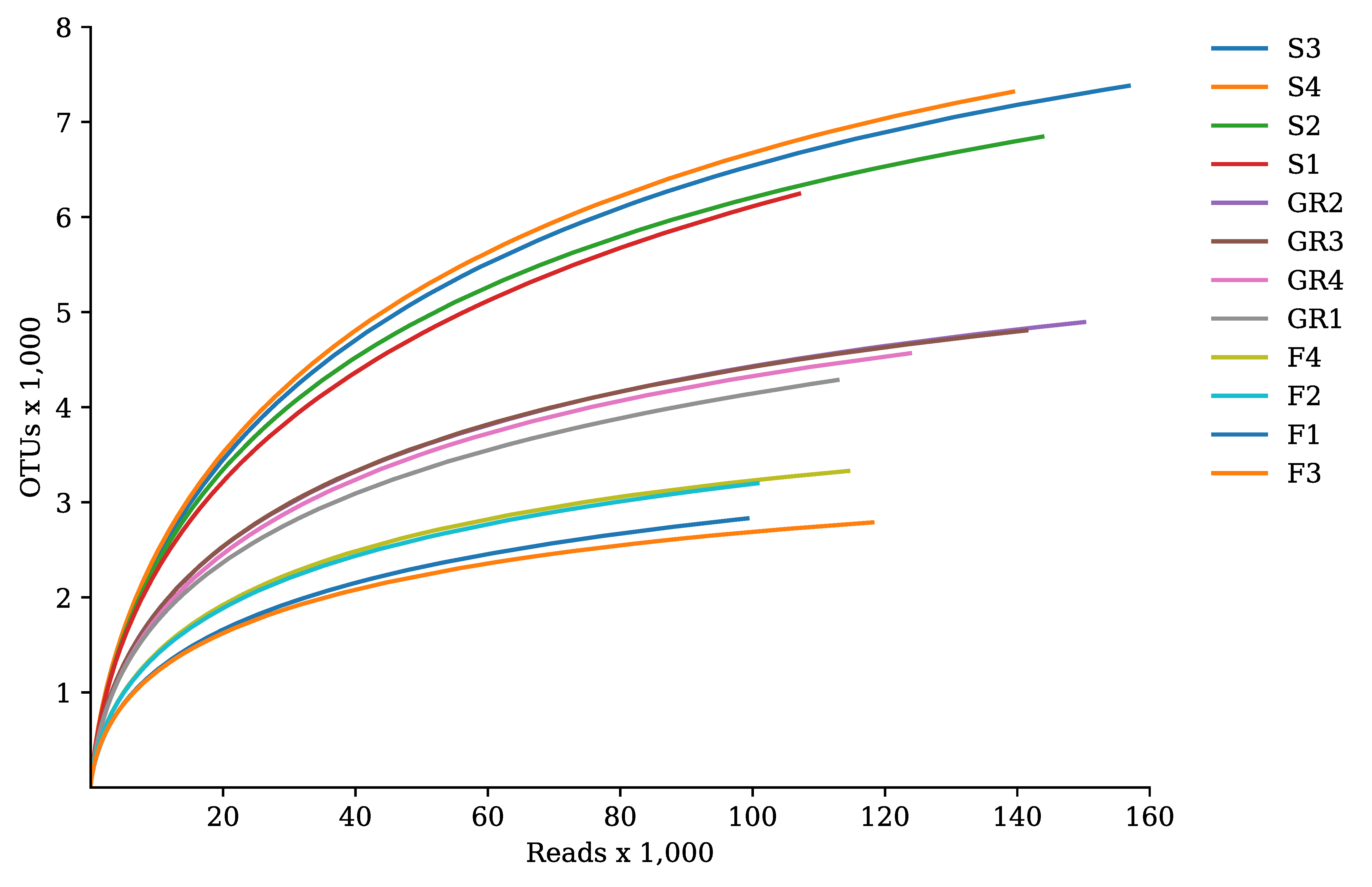

Supplement: S1 Fig — F = forest. GR = grassland. S = swamp. (TIF) [file pone.0243241.s001.tif]

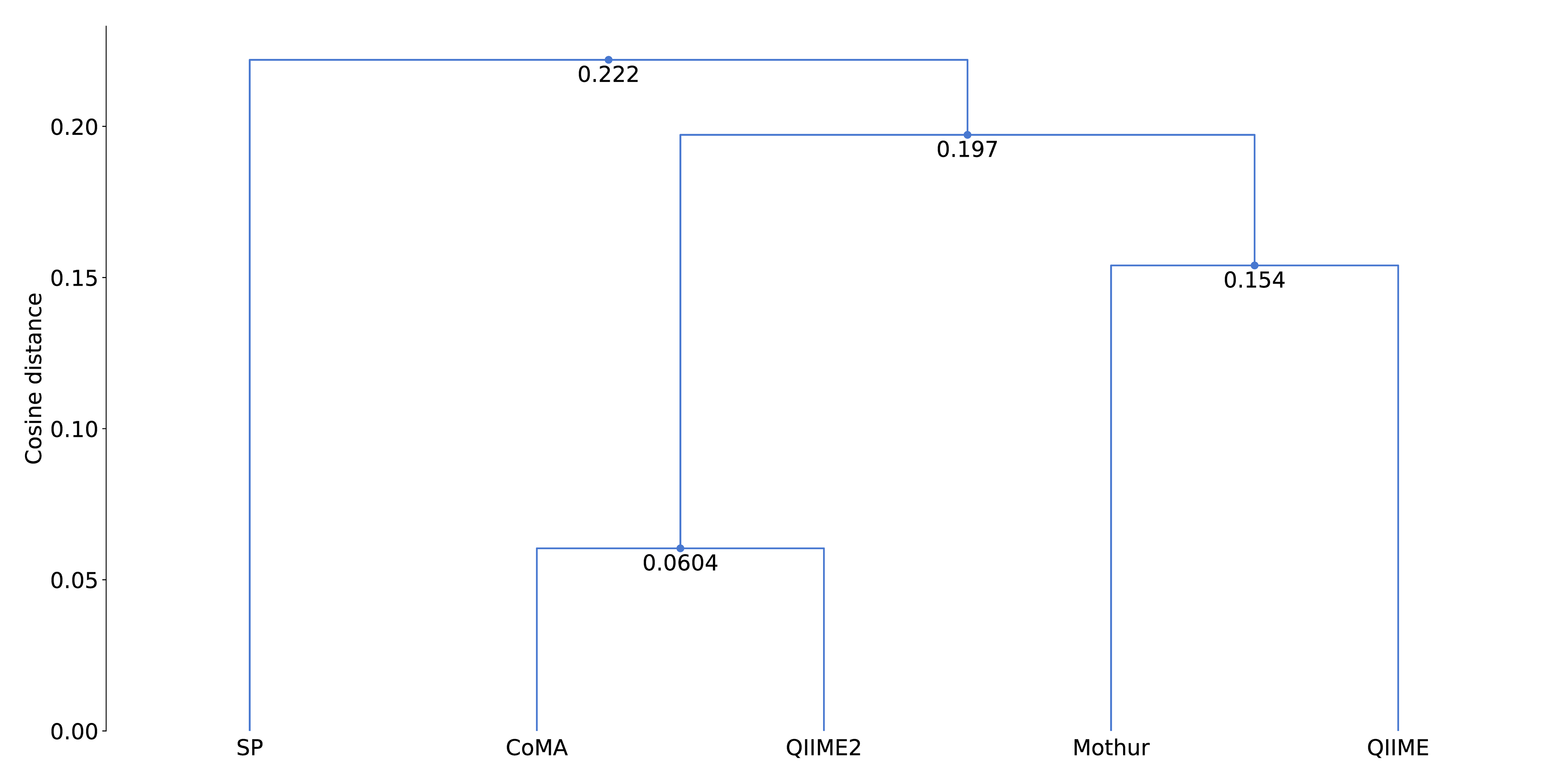

Supplement: S2 Fig — The dendrogram was calculated with the UPGMA method (unweighted pair group method with arithmetic mean) as bottom-up approach. (TIF) [file pone.0243241.s002.tif]

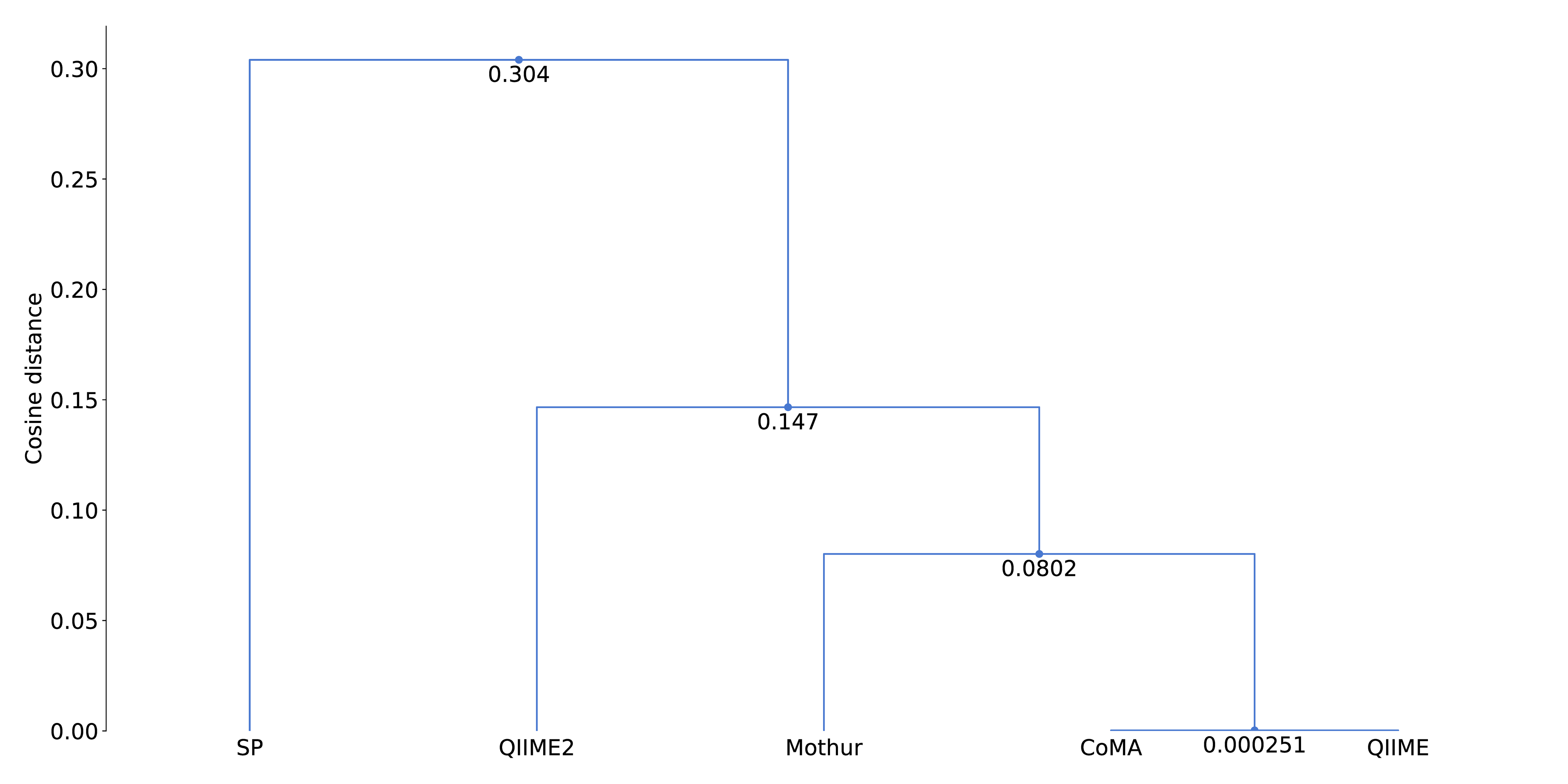

Supplement: S3 Fig — The dendrogram was calculated with the UPGMA method (unweighted pair group method with arithmetic mean) as bottom-up approach. (TIF) [file pone.0243241.s003.tif]

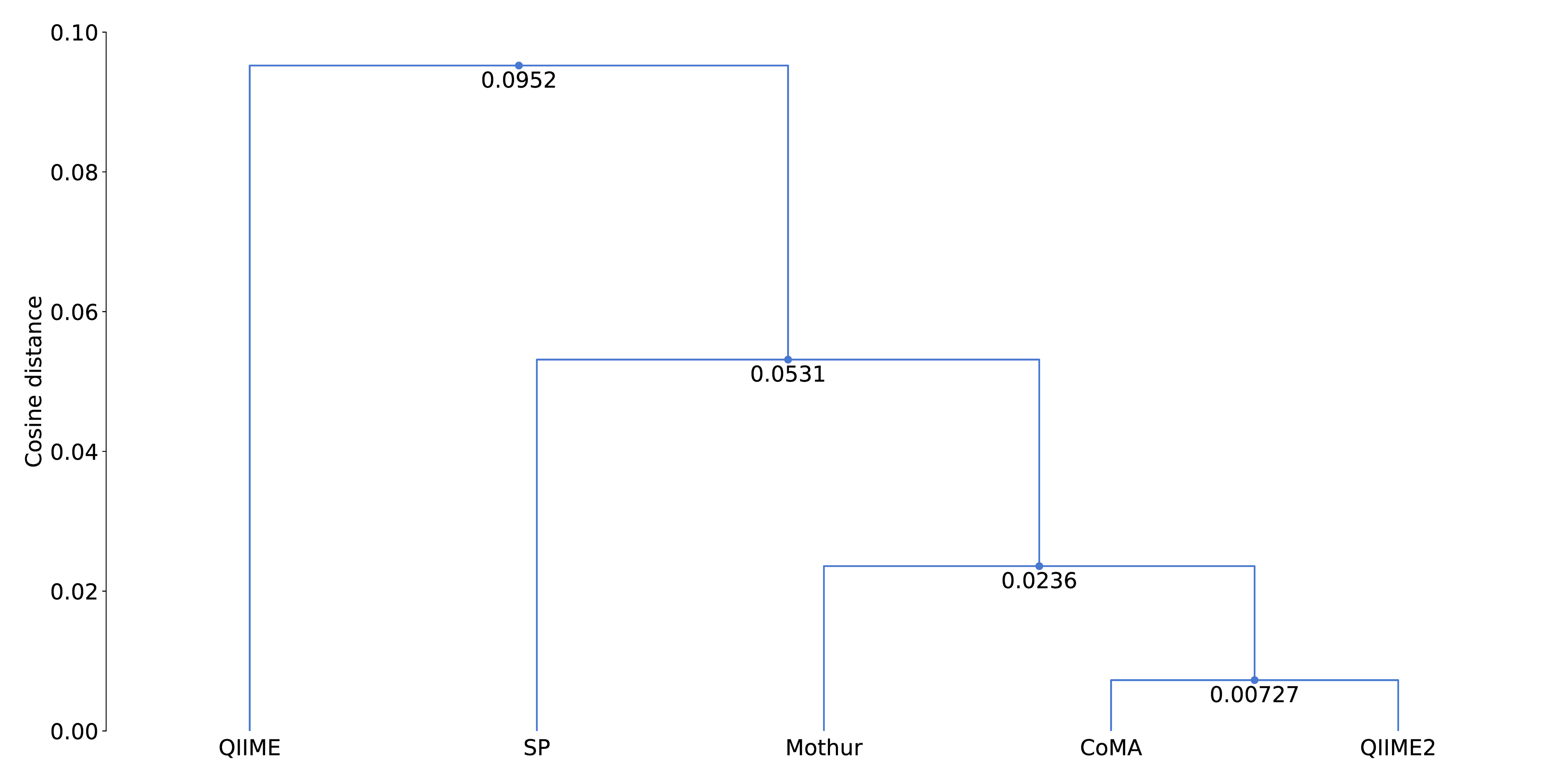

Supplement: S4 Fig — The dendrogram was calculated with the UPGMA method (unweighted pair group method with arithmetic mean) as bottom-up approach. (TIF) [file pone.0243241.s004.tif]

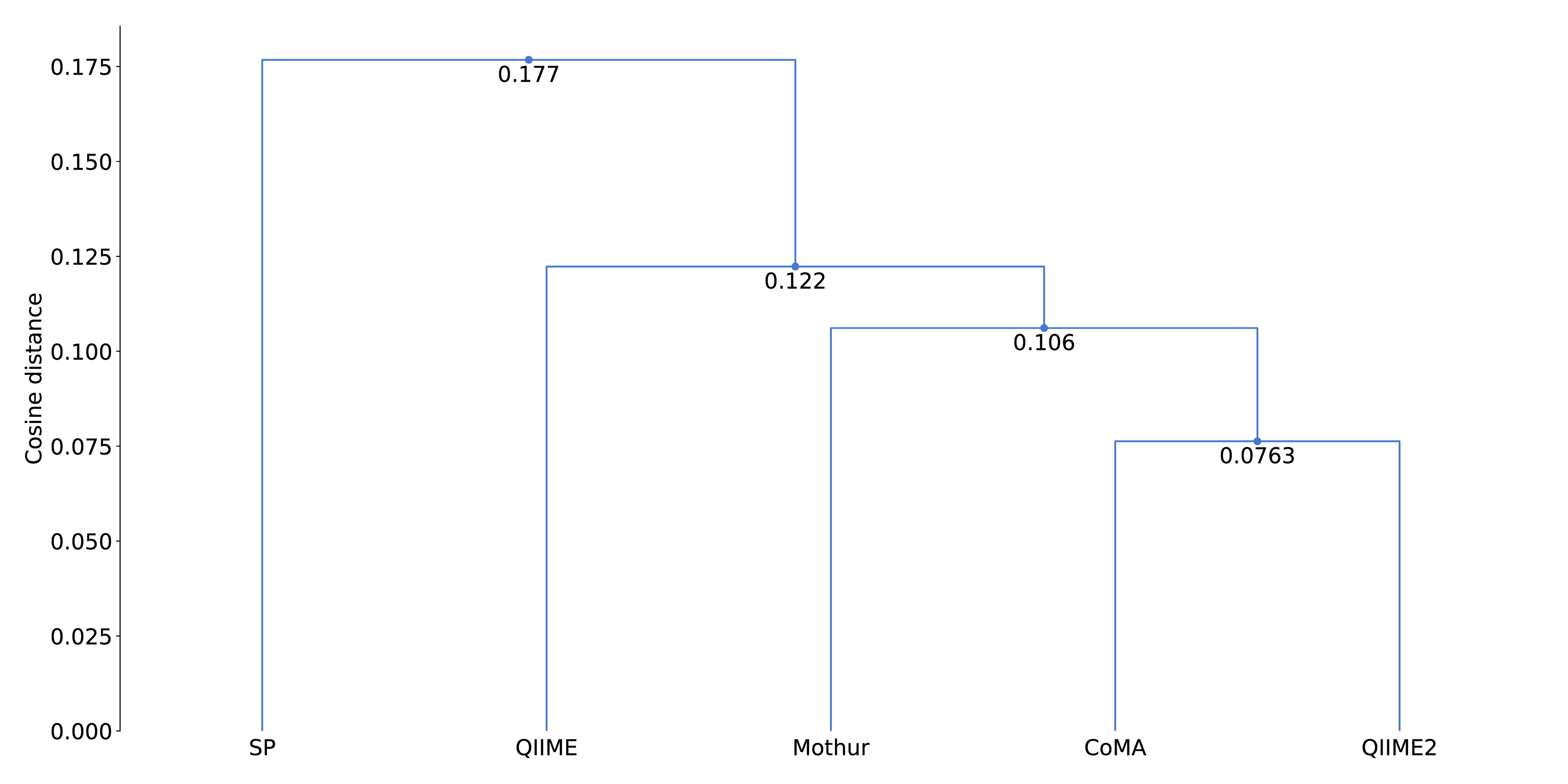

Supplement: S5 Fig — The dendrogram was calculated with the UPGMA method (unweighted pair group method with arithmetic mean) as bottom-up approach. (TIF) [file pone.0243241.s005.tif]

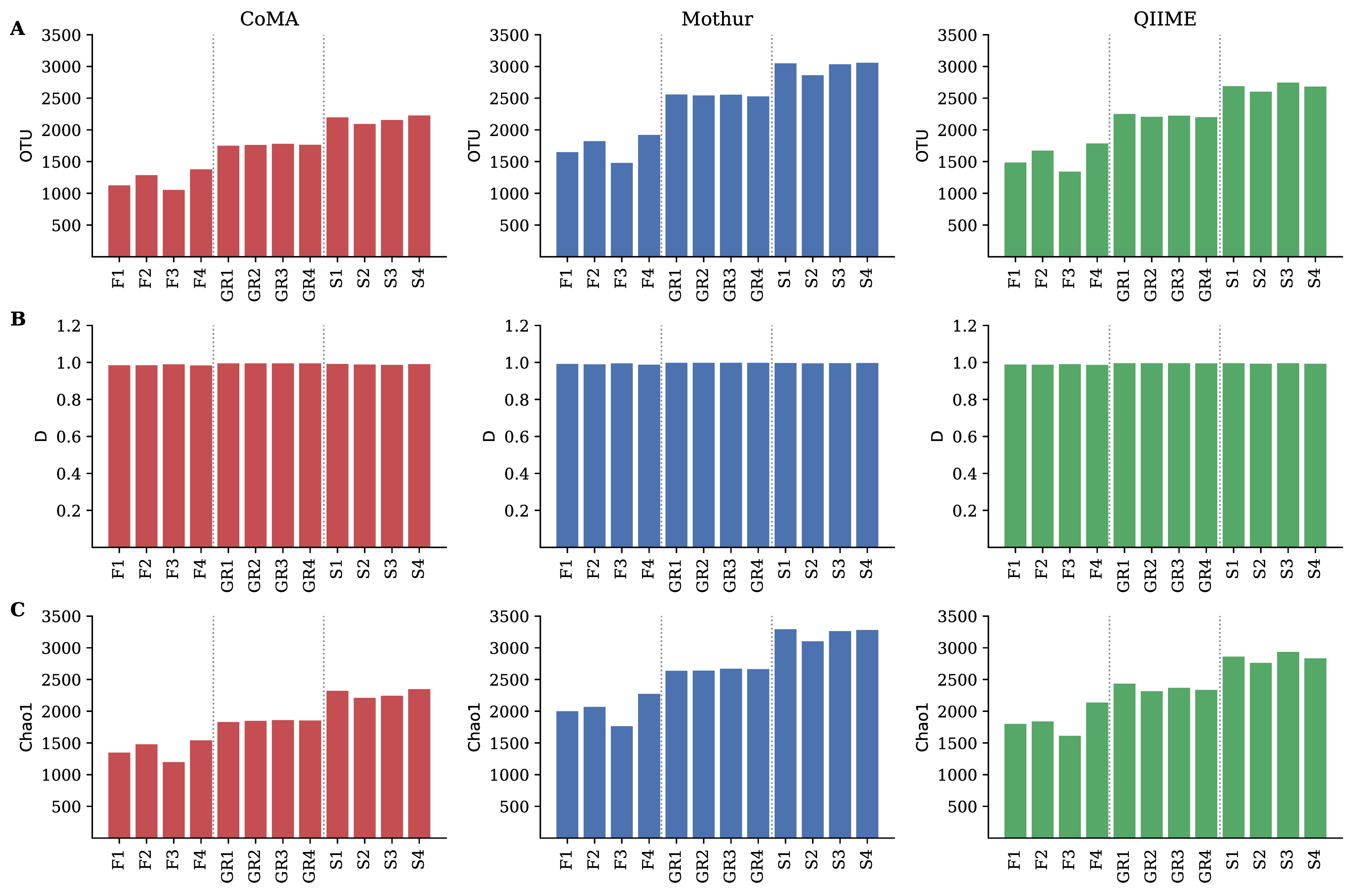

Supplement: S6 Fig — (A) Abundance (OTU, operational taxonomic unit), (B) Simpson diversity (D) and (C) Chao1 diversity of three different soils after sequencing data analysis with CoMA, Mothur and QIIME. Four replicates are shown for each habitat. F = forest. GR = grassland. S = swamp. (TIF) [file pone.0243241.s006.tif]
