# Supplementary material for: CoMA – an intuitive and user-friendly pipeline for amplicon-sequencing data analysis
Source: PLoS One. 2020 Dec 2;15(12):e0243241. doi: 10.1371/journal.pone.0243241 (PMC7710066; doi:10.1371/journal.pone.0243241)
Supplement: S1 Table — The table shows means ± standard deviation (n = 4). DM = dry matter. VS = volatile solids. EC = electrical conductivity. WHC = water holding capacity. (DOCX) [file pone.0243241.s007.docx]

| **Site** | **pH** | **DM**  **[%]** | **VS**  **[%]** | **EC**  **[µS cm^-1^]** | **N-NH_4_^+^**  **[µg g^-1^ DM]** | **N-NO_3_^-^**  **[µg g^-1^ DM]** | **Actual WHC**  **[%]** | **Max. WHC**  **[g H_2_O g^-1^ DM]** |
| --- | --- | --- | --- | --- | --- | --- | --- | --- |
| Grassland | **6.6** ± 0.0 | **66.6** ± 1.8 | **14.8** ± 1.8 | **190.8** ± 88.9 | **0.1** ± 0.0 | **3.1** ± 0.5 | **39.8** ± 3.7 | **1.3** ± 0.2 |
| Forest | **4.0** ± 0.3 | **73.0** ± 5.0 | **19.6** ± 3.5 | **99.6** ± 56.9 | **3.1** ± 0.4 | **0.8** ± 0.6 | **28.9** ± 4.7 | **1.4** ± 0.5 |
| Swamp | **6.7** ± 0.2 | **59.7** ± 3.2 | **11.2** ± 1.5 | **167.2** ± 82.1 | **1.2** ± 0.3 | **0.8** ± 0.2 ^a^ | **45.9** ± 3.9 | **1.6** ± 0.2 |

^a^ n = 3
