# Supplementary material for: CoMA – an intuitive and user-friendly pipeline for amplicon-sequencing data analysis
Source: PLoS One. 2020 Dec 2;15(12):e0243241. doi: 10.1371/journal.pone.0243241 (PMC7710066; doi:10.1371/journal.pone.0243241)
Supplement: S1 File — The file includes the results and a discussion of the microbial characteristics of three different soils (forest, grassland, swamp), which was determined with 16S amplicon sequencing. (PDF) [file pone.0243241.s008.pdf]

Soil data analysis with CoMA

Results

Alpha diversity analysis based on Archaea and Bacteria using three diversity indices, Shannon-Wiener, Chao1 and Simpson, revealed significant differences ($p < 0.001$) across the three soils (Fig 1). The highest Shannon-Wiener and Chao1 diversity was observed for the swamp samples, whereas the highest Simpson diversity was found for grassland. Species richness, in terms of OTU numbers, was greater for the swamp samples, followed by grassland and forest. Both, diversity and species richness were lower in the forest samples, irrespective of the applied index.

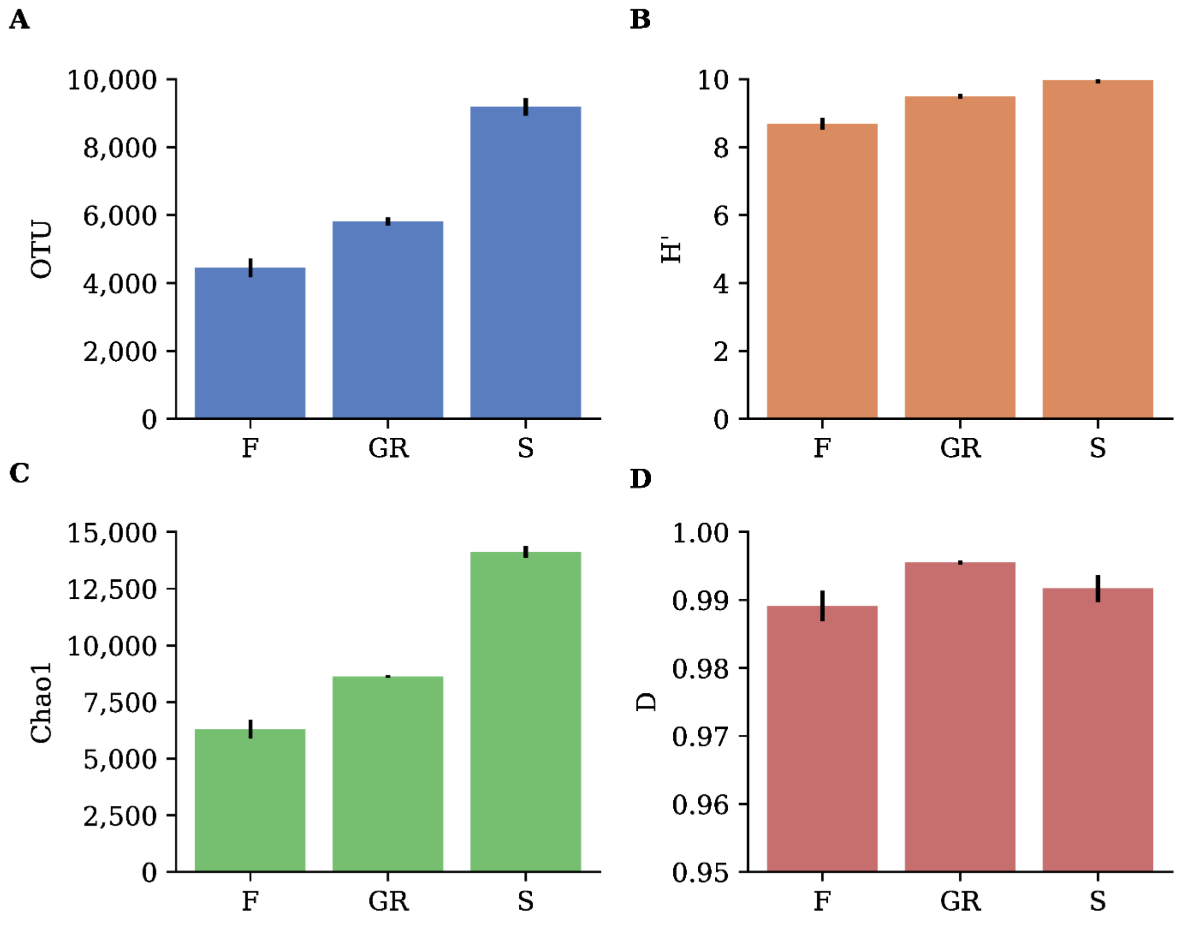

Fig 1. Species richness and alpha diversity of three different soils. (**A**) Species richness presented as operational taxonomic unit (OTU) count, and diversity analysis using three diversity indices: (**B**) Shannon-Wiener index, (**C**) Chao1 index and (**D**) Simpson index. Error bars indicate the standard deviation (n = 4). F = forest. GR = grassland. S = swamp.

Hierarchical cluster analysis using the Braycurtis metric showed a clear separation of samples belonging to the three soils (Fig 2). Therefore, it can be assumed that unique microbial communities developed in each habitat. However, the microbial consortia of swamp and grassland were closer related to each other (sequence similarity of 33%) than to the forest samples (10% similarity). All of the grassland and swamp replicates grouped close to each other (86% and 79% similarity, respectively) while the forest samples showed a significantly lower between-group similarity (57%).

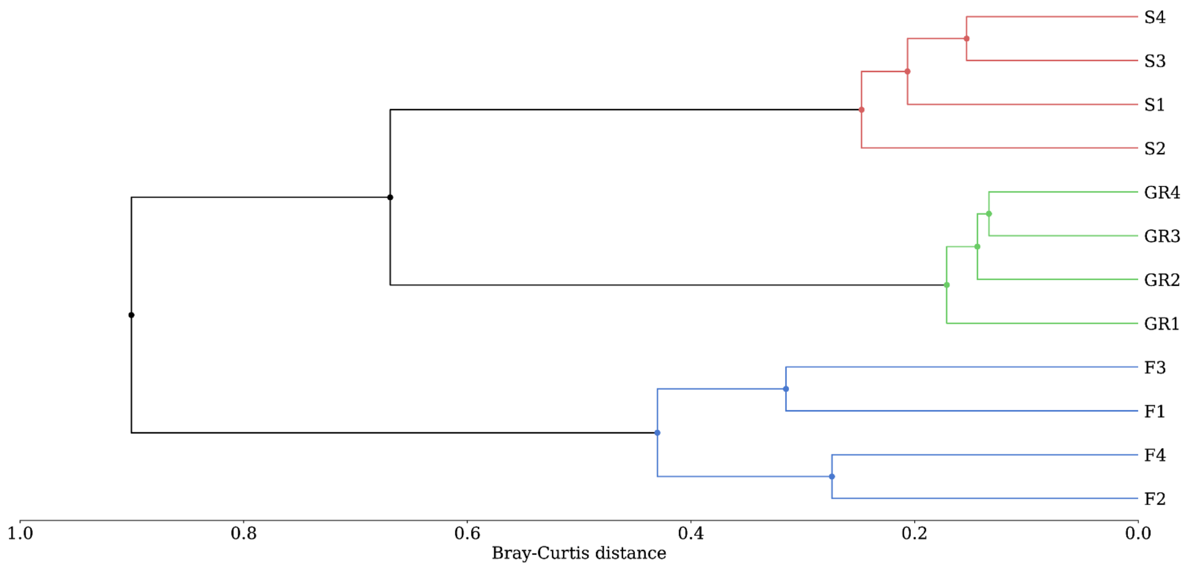

Fig 2. Cluster analysis performed on soil samples using the CoMA pipeline. The dendrogram was calculated with the UPGMA method (unweighted pair group method with arithmetic mean) as bottom-up approach. Data points were compared using the Braycurtis distance (n = 4). Different colors indicate groups with an internal similarity > 50%. F = forest. GR = grassland. S = swamp.

Looking at the bacterial community in detail, forest samples were clearly dominated by Chthoniobacteraceae (13%) and Xanthobacteraceae (10%; Fig 3). This latter family was also found in the two other habitats at a significantly lower abundance (3% and 2% in grassland and swamp, respectively). Chthoniobacteraceae also appeared in grassland (1%) but were below the detection limit in the swamp (< 1%). Other important families in the forest samples were Solibacteraceae (Subgroup 3; 5%), Acidobacteriaceae (Subgroup 1; 4%) and Gemmataceae (4%). These taxa were highly characteristic for the forest habitat and could not be found at comparable abundances in grassland or swamp.

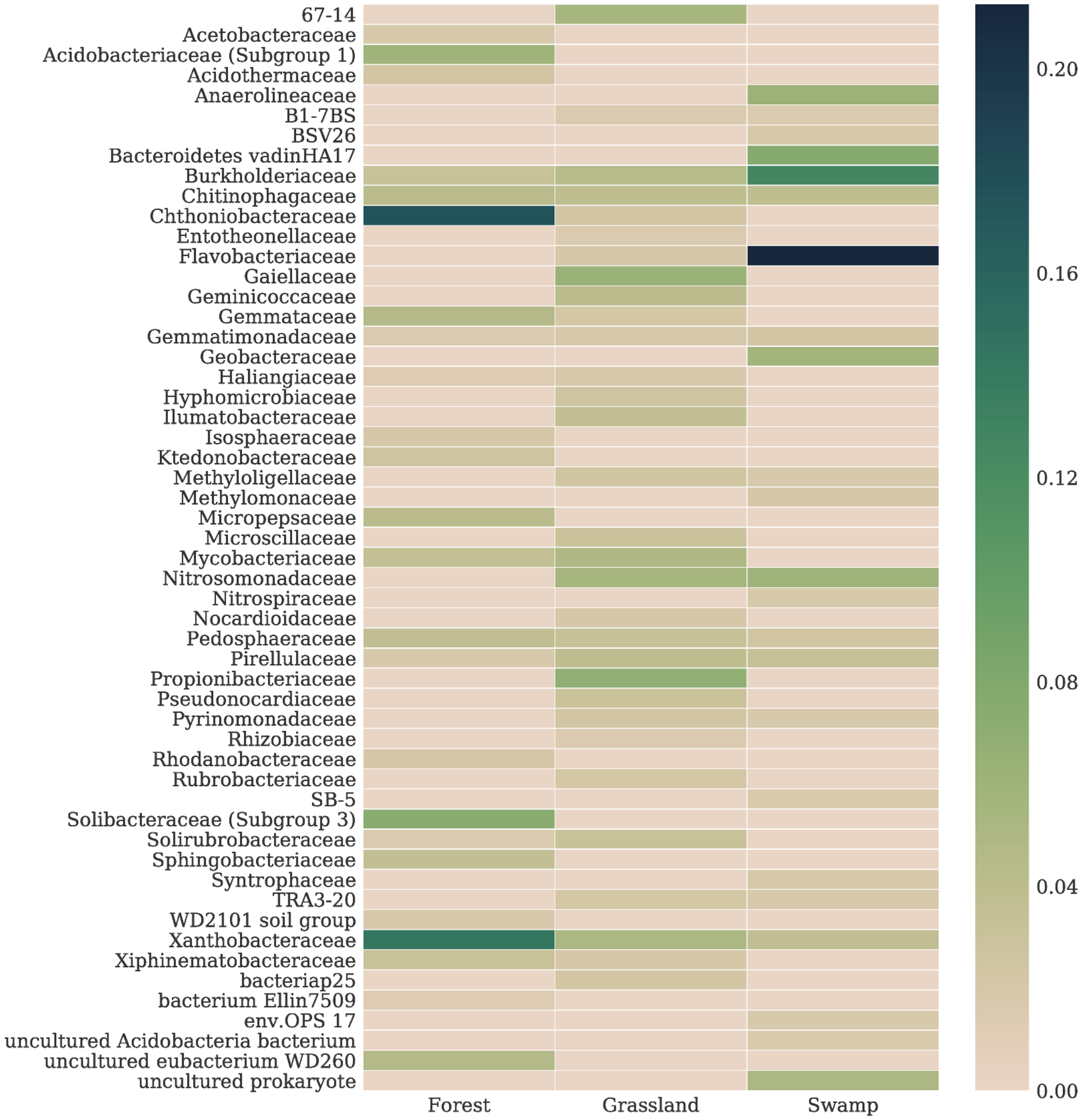

Fig 3. Heatmap showing the bacterial community of soil samples determined with the CoMA pipeline. Color code indicates relative abundance within each habitat. Families with an abundance > 1% are shown, unassigned taxa are excluded from depiction. Columns represent mean values for each investigated site (n = 4).

The grassland samples showed an even community structure with no clearly dominating taxa as observed for forest and swamp. Moreover, 31 different families were detected in grassland samples above the threshold of 1%. This was by far the highest number among all habitats (25 and 21 for forest and swamp, respectively), indicating a highly diverse and complex bacterial consortium. Dominant families in grassland samples were Propionibacteriaceae, Gaiellaceae, Nitrosomonadaceae and an uncultured Solirubrobacterales family (67-14) with an abundance of 4%, respectively. Swamp samples

were dominated by Flavobacteriaceae (12%) and Burkholderiaceae (7%). Both families were predominant in swamp compared to the two other habitats. Although Bacteroidetes vadinHA17 and Anaerolineaceae were less abundant (4% each), they were important indicators for the swamp soil since both families were neither found in forest, nor in the grassland soil.

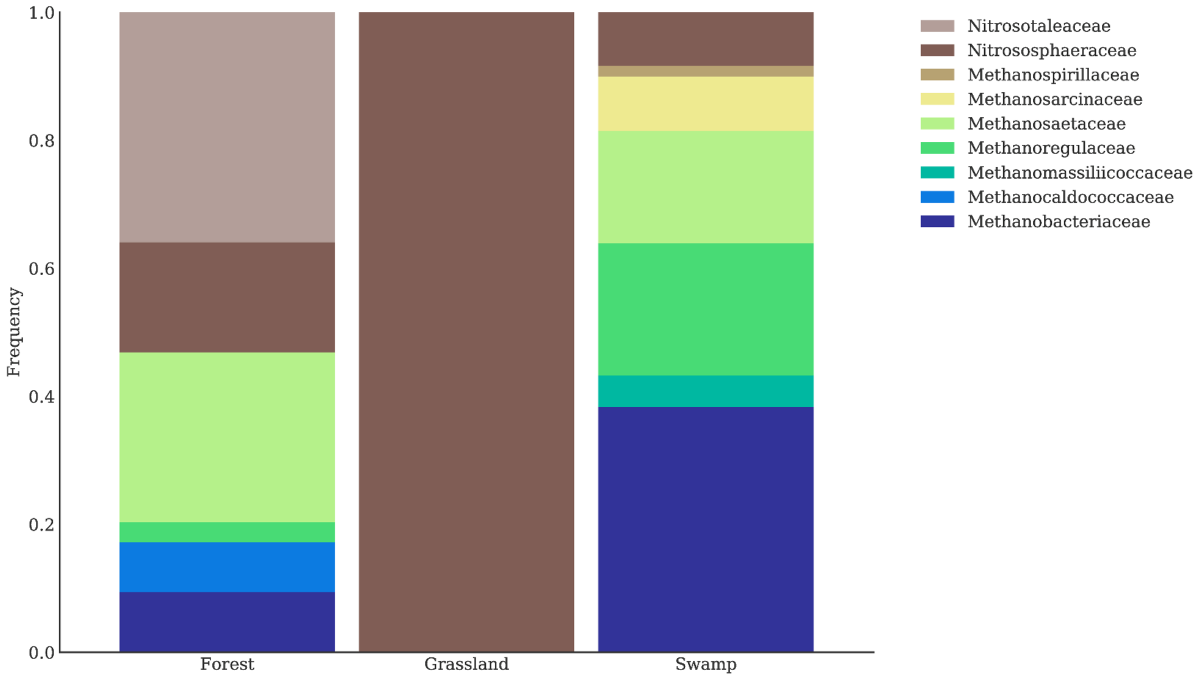

Fig 4. Archaeal soil community structure of three different soils. Data from 16S amplicon-sequencing and subsequent analysis using the CoMA pipeline. Families with abundance > 1% of the total archaeal community for each site are shown, while unassigned taxa are not included. Columns represent mean values for each investigated site (n = 4).

Taking a deeper look into the archaeal community structure of the forest samples, Nitrosotaleaceae and Methanosaetaceae were the most abundant ones (relative abundance of 36% and 27%, respectively; Fig 4) followed by Nitrososphaeraceae (17%). Methanobacteriaceae and Methanocaldococcaceae showed an abundance of 9% and 8%, respectively, while Methanoregulaceae accounted for at least 3% of all Archaea. In contrast, grassland was completely dominated by Nitrososphaeraceae (99%). Methanosaetaceae, Methanobacteriaceae, Methanocaldococcaceae, Methanomassiliicoccaceae and Methanoregulaceae were detected at an abundance < 1%. Methanobacteriaceae was the most abundant family in swamp samples (38%), followed by Methanoregulaceae (20%) and Methanosaetaceae (18%). Methanosarcinaceae and Nitrososphaeraceae both accounted for 8% of the classified reads, and

Methanomassiliicoccaceae for 5%. Methanospirillaceae (2%) and Methanocaldococcaceae (1%) were low in abundance but still detectable in the swamp samples.

Discussion

Although located in close proximity, each soil revealed a unique microbial community structure with different diversity and species richness. It is known from literature that both, the vegetation and the soil type are main drivers for microbial diversity in soils [1]. In this case, particularly the vegetation type, forest on the one hand and meadow (grassland, swamp) on the other hand, may have influenced the microbial community the most, since the climatic conditions and the parent material of the soils were the same for all sites. This assumption is also strengthened by the results of the cluster analysis where grassland- and swamp samples were much closer related to each other than to the forest. We assume that the soil pH, which was remarkably lower in the forest compared to the other two sites (S1 Table), was another crucial factor shaping microbial communities. Several authors, including Nacke et al. [2] and Zhalnina et al. [3], who further stressed out the importance of soil texture, already reported this. Ivanova et al. [4] stated that the soil acidity is the strongest driver in the formation of microbial communities and evokes fundamental changes at phylum level. According to the authors, the vegetation type, as second but slightly weaker factor, shapes the microbiota at lower taxonomic levels, e.g. order, family, or genus.

The high species richness and diversity in the swamp may be explained by the high content of organic compounds typically found in (partially) flooded soils. Moreover, this site has been fertilized with manure twice or thrice a year, which is expected to increase the soil organic matter content (OM). Tiedje et al. [5] raised the hypothesis that a high OM content may explain increased microbial diversity while Degens et al. [6] emphasized particularly the role of organic carbon. Despite being generally low in species richness and diversity, the forest soil showed a high variation in their microbial community structure among the replicates compared to grassland and swamp. This dissimilarity suggests a higher heterogeneity within microbial communities across the sampling area of 100 m^2 and may be explained by the different niches and soil heterogeneity typically found in forest soils [7]. The vegetation as well

as local physico-chemical characteristics at these local niches may play a key role in the development of individual microbial communities. This assumption may also apply for grassland and swamp; however, the differences between the replicates were much lower because the vegetational heterogeneity of the meadows was not comparable to the one in forest. High microbial heterogeneity in (forest) soils of the same type was also found at a microscopic scale and is assumed to be due to spatial separation and the establishment of microhabitats [8]. Individuals from these microhabitats are generally isolated and can only encounter other microbes through animal- and root transport or after rain events.

Chthoniobacteraceae as dominant family in forest soils is in line with findings of Vivanco et al. [9] who found this family to be dominant in the litter layer of a *Nothofagus* mixed forest. Chthoniobacteraceae are member of the phylum Verrucomicrobia (class: Spartobacteria, order: Chthoniobacterales) which is increasingly recognized as a crucial phylum of any soil ecosystem and typically accounts for 1.2-10.9% of the total bacteria in soil [10]. The family Xanthobacteraceae is another core member of forest soils [11-13]. For grassland, Propionibacteriaceae were among the key families. This is remarkable since these Gram$^+$ bacteria are anaerobes [14], typically found in the digestive tract of animals [15] but not in unfertilized soil systems. The investigated site, however, was not fertilized for ten years and Propionibacteriaceae had not been expected after such a long time. Therefore, we assume that they were introduced to the ecosystem by manure application and persisted in appropriate niches. It remains, however, unclear why Propionibacteriaceae were not found in the swamp where manure is amended regularly and the fluctuating water level causes, at least partially, anaerobic zones. Nitrosomonadaceae, another abundant family in the grassland samples, contains nitrifying bacteria such as *Nitrosomonas* and *Nitrosospira*, which are involved in the oxidation of ammonia to nitrate in the presence of oxygen [16]. Their emergence is in line with the fact that a low ammonium content and an accumulation of nitrate were found in this habitat (S1 Table). Nitrosomonadaceae were also detected in the swamp; however, occasional oxygen limitations might have hindered the nitrification process and may explain higher ammonium and lower nitrate levels compared to the grassland. Beyond that, the organic fertilization of the swamp soil might also have influenced the ammonium- and nitrate levels, both directly and indirectly. Other characteristic taxa in the grassland were members of the Actinobacteria (e.g.

Gaiellaceae, Solirubrobacterales), which are known as key players in terrestrial and aquatic ecosystems [17, 18].

Low nitrate levels in the swamp may be alternatively explained with the distinctive occurrence of *Flavobacterium*. Some of these microbes are aerobically living denitrifiers (e.g. *Flavobacterium denitrificans* [19]) that convert nitrate to molecular nitrogen under anaerobic conditions and may explain why no nitrate accumulation took place in the swamp soil. Some denitrifiers are also known within the family Burkholderiaceae, e.g. *Ralstonia eutropha* (syn.: *Alcaligenes eutrophus* [20]), which were found in the swamp and in lower abundance in the forest and the grassland soil samples. Burkholderiaceae is a very diverse family and includes nitrogen-fixing bacteria like *Burkholderia vietnamiensis* [21]. These form close symbioses with plants and convert gaseous dinitrogen to ammonia that becomes available to the plant [22, 23]. Anaerolineaceae, which were exclusively detected in the swamp, are strict anaerobes and their presence is indicative of arbuscular mycorrhizal interactions between fungi and plant roots [24].

References

1. Garbeva P, Van Veen JA, Van Elsas JD. Microbial diversity in soil: selection of microbial populations by plant and soil type and implications for disease suppressiveness. Annu. Rev. Phytopathol. 2004; 42: 243-270. doi: 10.1146/annurev.phyto.42.012604.135455
2. Nacke H, Thürmer A, Wollherr A, Will C, Hodac L, Herold N, et al. Pyrosequencing-based assessment of bacterial community structure along different management types in German forest and grassland soils. PLOS One. 2011; 6: e17000. doi: 10.1371/journal.pone.0017000
3. Zhalnina K, Dias R, de Quadros PD, Davis-Richardson A, Camargo FA, Clark IM, et al. Soil pH determines microbial diversity and composition in the park grass experiment. Microb. Ecol. 2015; 69: 395-406. doi: 10.1007/s00248-014-0530-2
4. Ivanova E, Pershina E, Kutovaya O, Sergalieva NK, Nagieva A, Zhiengaliev A, et al. Comparative Analysis of Microbial Communities of Contrasting Soil Types in Different Plant Communities. Russ. J. Ecol. 2018; 49: 30-39. doi: 10.1134/S106741361801006X

5. Tiedje J, Cho J, Murray A, Treves D, Xia B, Zhou J. Soil Teeming with Life: New Frontiers for Soil Science. In: Rees RM, Ball BC, Campbell CD, Watson CA, editors. Sustainable Management of Soil Organic Matter. 2001. pp. 393-412.
6. Degens BP, Schipper LA, Sparling GP, Vojvodic-Vukovic M. Decreases in organic C reserves in soils can reduce the catabolic diversity of soil microbial communities. Soil Biol. Biochem. 2000; 32: 189-196. doi: 10.1016/S0038-0717(99)00141-8
7. Bruckner A, Kandeler E, Kampichler C. Plot-scale spatial patterns of soil water content, pH, substrate-induced respiration and N mineralization in a temperate coniferous forest. Geoderma. 1999; 93: 207-223. doi: 10.1016/S0016-7061(99)00059-2
8. Nannipieri P, Ascher J, Ceccherini M, Landi L, Pietramellara G, Renella G. Microbial diversity and soil functions. Eur. J. Soil Sci. 2003; 54: 655-670. doi: 10.1046/j.1351-0754.2003.0556.x
9. Vivanco L, Rascovan N, Austin AT. Plant, fungal, bacterial, and nitrogen interactions in the litter layer of a native Patagonian forest. PeerJ. 2018; 6: e4754. doi: 10.7717/peerj.4754
10. Sangwan P, Chen X, Hugenholtz P, Janssen PH. Chthoniobacter flavus gen. nov., sp. nov., the first pure-culture representative of subdivision two, Spartobacteria classis nov., of the phylum Verrucomicrobia. Appl. Environ. Microb. 2004; 70: 5875-5881. doi: 10.1128/AEM.70.10.5875-5881.2004
11. Khodadad CL, Zimmerman AR, Green SJ, Uthandi S, Foster JS. Taxa-specific changes in soil microbial community composition induced by pyrogenic carbon amendments. Soil Biol. Biochem. 2011; 43: 385-392. doi: 10.1016/j.soilbio.2010.11.005
12. Uroz S, Oger P, Tisserand E, Cébron A, Turpault M-P, Buée M, et al. Specific impacts of beech and Norway spruce on the structure and diversity of the rhizosphere and soil microbial communities. Sci. Rep. 2016; 6: 27756. doi: 10.1038/srep27756
13. Vik U, Logares R, Blaalid R, Halvorsen R, Carlsen T, Bakke I, et al. Different bacterial communities in ectomycorrhizae and surrounding soil. Sci. Rep. 2013; 3: 3471. doi: 10.1038/srep03471
14. Goldschmidt P, Ferreira CC, Degorge S, Benallaoua D, Boutboul S, Laroche L, et al. Rapid detection and quantification of Propionibacteriaceae. Brit. J. Ophthalmol. 2009; 93: 258-262. doi: 10.1136/bjo.2008.146639

15. Borriello S. Microbial flora of the gastrointestinal tract. In: Hill MJ, editor. Microbial metabolism in the digestive tract. 1986. pp. 2-16.

16. Braker G, Conrad R. Diversity, structure, and size of N2O-producing microbial communities in soils—what matters for their functioning?. Adv. Appl. Microbiol. 2011; 75: 33-70. doi: 10.1016/B978-0-12-387046-9.00002-5

17. Liu X, Cong J, Lu H, Xue Y, Wang X, Li D, et al. Community structure and elevational distribution pattern of soil Actinobacteria in alpine grasslands. Acta Ecol. Sin. 2017; 37: 213-218. doi: 10.1016/j.chnaes.2017.02.010

18. Ward AC, Bora N. Diversity and biogeography of marine actinobacteria. Curr. Opin. Microbiol. 2006; 9: 279-286. doi: 10.1016/j.mib.2006.04.004

19. Horn MA, Ihssen J, Matthies C, Schramm A, Acker G, Drake HL. Dechloromonas denitrificans sp. nov., Flavobacterium denitrificans sp. nov., Paenibacillus anaericanus sp. nov. and Paenibacillus terrae strain MH72, N2O-producing bacteria isolated from the gut of the earthworm Aporrectodea caliginosa. Int. J. Syst. Evol. Micr. 2005; 55: 1255-1265. doi: 10.1099/ijs.0.63484-0

20. Siddiqui R, Warnecke-Eberz U, Hengsberger A, Schneider B, Kostka S, Friedrich B. Structure and function of a periplasmic nitrate reductase in Alcaligenes eutrophus H16. J. Bacteriol. 1993; 175: 5867-5876. doi: 10.1128/jb.175.18.5867-5876.1993

21. Estrada-De Los Santos P, Bustillos-Cristales R, Caballero-Mellado J. *Burkholderia*, a genus rich in plant-associated nitrogen fixers with wide environmental and geographic distribution. Appl. Environ. Microbiol. 2001; 67: 2790-2798. doi: 10.1128/AEM.67.6.2790-2798.2001

22. Elliott GN, Chen WM, Chou JH, Wang HC, Sheu SY, Perin L, et al. *Burkholderia phymatum* is a highly effective nitrogen-fixing symbiont of *Mimosa spp.* and fixes nitrogen ex planta. New Phytol. 2007; 173: 168-180. doi: 10.1111/j.1469-8137.2006.01894.x

23. Reis V, Estrada-De Los Santos P, Tenorio-Salgado S, Vogel J, Stoffels M, Guyon S, et al. Burkholderia tropica sp. nov., a novel nitrogen-fixing, plant-associated bacterium. Int. J. Syst. Evol. Micr. 2004; 54: 2155-2162. doi: 10.1099/ijs.0.02879-0

24. Rodríguez-Caballero G, Caravaca F, Fernández-González A, Alguacil M, Fernández-López M, Roldán A. Arbuscular mycorrhizal fungi inoculation mediated changes in rhizosphere bacterial

community structure while promoting revegetation in a semiarid ecosystem. Sci. Total Environ. 2017; 584: 838-848. doi: 10.1016/j.scitotenv.2017.01.128
