# Supplementary material for: CoMA – an intuitive and user-friendly pipeline for amplicon-sequencing data analysis
Source: PLoS One. 2020 Dec 2;15(12):e0243241. doi: 10.1371/journal.pone.0243241 (PMC7710066; doi:10.1371/journal.pone.0243241)
Supplement: Attachment — Submitted filename: Response to Reviewers.docx [file pone.0243241.s009.docx]

***PONE-D-20-10229R1***

***Editors/Reviewers' comments***

Below are the responses to the Editors/Reviewers’ comments. The line numbers we are referring to are those of the revised manuscript (“Revised Manuscript with Track Changes.docx”).

**Reviewer #1:**

***The sentence "It seems that QIIME2 for some reason did not properly process the data in this situation" is still ambiguous. While the authors have observed that the tool didn't work well in a specific context, any interpretation is subject to caution. Maybe this processing is "proper" to avoid false discoveries, for instance. If there is some actual problem on the path to the results, it's valid information which should be given (for example, any problem or error returned to the user during the processing, intermediate or output files lacking or incomplete, presence of an algorithmic flaw which explains the behaviour, etc..., anything factual and specific). If there is no factual justification, then please only retain the part about the missing 25% and delete the comment about proper processing.***

DONE. We removed this statement completely, since there was no clear evidence for a specific problem in course of the data processing. Please see line 665.

***There seem to have been a misunderstanding about other pipelines, which don't need to be exhaustively examined in detail. Meanwhile, providing a list of directly relevant pipelines and if possible a brief situating comment is often a primary expectation from the discussion part of a method article. Please include at the very least a list of other directly relevant pipelines, and as far as possible situate them briefly relatively to CoMA. For instance, there might be functionalities which are offered by CoMA and not by others.***

DONE. We now added a new table (Table 1) showing a selection of existing software packages for amplicon sequencing data analysis. The table includes important characteristics of the tools as well as drawbacks relative to CoMA.

***QC is an essential, not redundant part of the methods. Please restore this part of the text and provide the required details about how it was carried out. It's fine even if it's just a visual review, or a custom QC analysis.***

DONE. We totally agree that QC and quality filtering are crucial for NGS data analysis and that this information always needs to be provided in a publication! In course of the revision process (first round), we shortened the Material & methods section, but just by removing repetitive passages. We are convinced that each important information, particularly in terms of quality filtering, is still provided. The chapters on soil data analysis are referring now to the description of the mock data analysis (*mock-13*) since the protocols and settings were mostly the same. We slightly modified these text passages now in order to state clearly that all the relevant information is provided in the previous sections; please see lines 379-380, 391-392 and 395-396.

***Other comments have been addressed or justified appropriately.***

Thank you very much for your constructive inputs (also during the first round of revision), which notably improved the quality of our manuscript!
