# Supplementary material for: CoMA – an intuitive and user-friendly pipeline for amplicon-sequencing data analysis
Source: PLoS One. 2020 Dec 2;15(12):e0243241. doi: 10.1371/journal.pone.0243241 (PMC7710066; doi:10.1371/journal.pone.0243241)
Supplement: Attachment — Submitted filename: Response to Reviewers.docx [file pone.0243241.s010.docx]

**PONE-D-20-21940**

***Reviewers' comments***

Below are the responses to the Reviewers’ comments. The line numbers we are referring to are those of the revised manuscript (“Revised Manuscript with Track Changes.docx”).

***Academic Editor***

***Please ensure that your manuscript meets PLOS ONE's style requirements, including those for file naming.***

DONE. We conscientiously revised the manuscript in order to meet the journal’s style requirements. Below, there is a summary of the most important changes:

- Title changed to sentence chase
- Removed telephone and fax numbers of the corresponding author
- Removed the chapter “Author summary”
- Removed the labelling of the headings
- The term “supplementary material” was removed when referencing supplementary figures and tables
- Removed the chapter “Supplementary Material”

***Reviewer #1***

***The manuscript by Hupfauf et al. describes COMA, a pipeline designed to the analysis of metabarcoding data. The paper is overall well written. However, there are some flaws that need to be addressed as illustrated below.***

Thank you for sharing your constructive feedback! The performed changes based on your suggestions have truly increased the quality of this manuscript!

***Introduction:***

***L77-78 The list of reference databases should be improved by including MIDORI, PR2 DB and ITSoneDB. Moreover authors should discuss the fact that actually both Greengenes and RDP are outdated.***

DONE. We have included the three additional databases, and provided now the year of the last update for the Greengenes and RDP databases in order to clarify that these databases are not up to date anymore; please see lines 68-71.

***L 88. Authors should discuss the differences between OTU and ASV. They cite the paper by Callahan et al 2017 in Discussion but they did not describe the technical differences between the two approaches.***

DONE. We have compared OTU and ASV approaches now in more detail, please see lines 76-81.

***L 126 454 was terminated in 2016.***

DONE. Changed accordingly, please see line 116.

***Table 1: Add PEMA***

DONE. We have added the PEMA pipeline to Table 1.

***M&M***

***L 170-172 Authors stated in COMA a denoising and error correction step is implemented by using Lotus. It is not clear what they mean with “denoising and error correction”. Literally denoising is the procedure applied to remove the noise introduced by amplification and sequencing, as stated by Quince et al in the paper describing AmpliconNoise. Actually, the denoising procedures are strictly related to the used sequencing platform as well as different sequencing approaches are characterized by different error profiles. In the introduction, authors declare COMA is able to analyze Illumina, PacBio and Roche 454 data, consequently if a denoising step is implemented in the pipeline, it must take into account the different peculiarities of these sequencing approaches. Authors should clarify this point.***

The quality filtering is done using LotuS, precisely the sdm tool. Regarding the LotuS publication (<https://doi.org/10.1186/2049-2618-2-30>), sdm “… filter(s) input sequences after average quality, accumulated error over the sequence, quality in a freely definable window and remove 5′ low-quality bases filtered for these criteria. Furthermore, sequences are filtered for min/max length, ambiguous nucleotides, max barcode and primer errors, polynucleotide runs, and trimmed for adapter sequences, if present.”. The reviewer is right in the sense that the filtering settings largely depend on the sequencing platform; hence, LotuS provides pre-adjusted sdm setting files for MiSeq, HiSeq, 454 and PacBio. The newest version of the CoMA pipeline now asks the user, which sequencing platform was used, and selects the corresponding sdm file for the sequence filtering process.

***L 176 The RDP-classifier is a Bayesian classifier inferring the posterior probability based on the known kmer distribution in genera and the observed one. Consequently it do not perform any alignment. Moreover it is not described how blast alignment is performed (default options?) and if it is filtered according to the observed similarity.***

DONE. We slightly rephrased this sentence in order to prevent any misunderstandings, please see lines 169-172. Blast is performed using the LotuS package with the default LotuS options. More details can be found in the LotuS publication (<https://doi.org/10.1186/2049-2618-2-30>).

***L 230-343 My major concerns are related to both the choice and the analysis of Mock data. Concerning the selected mocks: i) the inclusion of a mock with few dominant taxa and other less abundant taxa could recapitulate a real condition in several cases; ii) in consideration that 454 has been discontinued the choice of a ITS mock obtained by 454 sequencing is not very useful nowadays.***

Thank you very much for your feedback on our Mock analysis! The selection of suitable Mock datasets was done according to the recommendations of a previous reviewer. We tried to cover as much variation as possible in terms of diversity, marker genes, and sequencing platforms. By doing that, we wanted to demonstrate how CoMA performs when analyzing different types of data, always in comparison to established tools. It would indeed be interesting to analyze much more different Mock datasets (e.g. highly uneven distributed taxa, functional gene markers, PacBio data, etc.). However, this additional information would further extend the scope of this MS and it would be better to address it within another publication.

***In the following some comments concerning the analysis of mock data. First of all by taking into account for each mock the mixed strains are known, it is possible to infer the expected amplicon sequences, by performing an in silico PCR on the reference genomes. This would also allow to measure the intra- and inter- generic sequence variability and compare the observed OTUs/ASVs with the expected sequences.***

This is indeed an interesting approach! We are gladly taking this idea for our upcoming study and expect to gain even more information out of our analysis, particularly in terms of sequence variability.

***Moreover, it is not completely clear how taxonomic assignment was performed with the different pipelines. While in COMA an alignment based approach was used (see comments above for related issues), in QIIME the author used the assign-taxonomy.py script which implements 5 different taxonomic assignment methods (including blast), but they do not indicate the used one (uclust is the default).***

DONE. In QIIME, taxonomic assignment was done with “uclust”. In order to avoid any uncertainty, we have added this information; please see lines 301-303.

***The same issue is related to the taxonomic classification in Mothur while in QIIME2 a naïve-bayesian classifier trained on the same target region was used. Actually it seems different methods for taxonomic classification were applied and this may influence the comparison of the results.***

We are aware of the different algorithms that are implemented in the compared tools. Our approach, however, was to compare CoMA with the suggested procedures (SOPs) of Mothur, QIIME and QIIME2. In the end, all tools should depict the mock community as precisely as possible, irrespective of the applied algorithms (what they did quite well).

***Moreover, the ASV inference pipeline implemented in QIIME2 is deeply influence by the used filtering parameters. Have the authors used the default parameters or have adjusted them according to the overall data quality? Finally, authors should indicate the versions of the used tools.***

DONE. As for QIIME and Mothur, we also used the recommended settings for the QIIME2 analysis from the official SOP documentation. We are providing the applied versions of Mothur, QIIME and QIIME2 now in the manuscript; please see lines 260, 292 and 311.

***Figure 2, 3 and 4 I suggest to change the colors in order to improve the plots.***

Figures 2-4 show bar charts using the color code that is currently used by CoMA for all graphics. Hence, we would like to keep the colors as they are, in order to demonstrate the CoMA style continuously throughout the whole paper. However, we are aware that sometimes users might have very specific color-requirements. Therefore, we have decided to implement an option for selecting an individual color map for all plots in CoMA within the next update of the pipeline.

***In general, I find the manuscript is too long and not focused. Indeed, it includes two messages: the description of a novel pipeline for DNA metabarcoding analysis, and an analysis of real data of soil samples. Maybe the Authors can consider to reduce the part concerning real data to the comparative aspects, moving the microbiome analysis of soil samples and its discussion to a different manuscript focused on this matter.***

Thank you for this critical comment! We had discussed about this issue when starting to write the paper. After weighing the pros and cons of combining a method paper with the analysis of real data, we decided to keep it combined because we are convinced that it is beneficial for the majority of CoMA users to see the pipeline being demonstrated on a real dataset (including an extensive interpretation and discussion of the results). Moreover, we aimed at providing any relevant information on CoMA in a single extensive publication rather than splitting it up into two separate papers, one solely describing the pipeline and one demonstrating its functionality based on real data.

***Reviewer #2***

***This manuscript reported a pipeline CoMA for amplicon-sequencing data analysis. It reported that comparing to other command line tools, the pipeline CoMA's advantages are much easier, more user-friendly, compatible with any common operating system, and good performance compared.***

Thank you very much for reviewing our manuscript and for sharing your constructive feedback! Please find below our detailed response to each individual point.

***Major comments:***

***1. The CoMA is distributed by a 5.77GB VirtualBox .ova file. I cannot test it on my remote lab server because it doesn't have GUI to use VirtualBox. Due to COVID19, I couldn't access my high performance desktop computer in my office. I couldn't test it on my home laptop because of CoMA's large disk space requirement (50GB). Hence, I am sorry that I didn't test whether it can be the installed and work correctly. Hope other reviewers did that. In fact, I don't think it's an advantage to use virtual machine image for distributing CoMA. I think Docker is a much better and easier solution for both software maintainers and users.***

It is unfortunate that you were not able to test CoMA personally! Please feel free to test it later, your feedback is highly appreciated! We are currently working on alternative ways of installing CoMA in the near future. This will include a Docker image, a direct Linux installer, as well as a Singularity container, which will also allow the usage of CoMA on a HPC cluster system. We are very confident to provide all of them within the next two or three weeks and will advertise these novel features on the CoMA homepage.

***2. As I didn't run CoMA by myself, so I didn't see it's graphical interface. However, according to the description in the manuscript and CoMA's manual online, I don't think its GTK+ dialog GUI is better than a command line interactive mode. I don't think command line is hard to use, and I don't think CoMA is easier and user-friendly than command line tools.***

CoMA was initially coded as command line-based pipeline. After having been extensively tested (including by entry-level users), we decided to include the GTK+ dialogs taking their feedback into account. According to our testers, this considerably facilitated the workflow. Apart from that, there are also other aspects making CoMA user-friendly and easy to use for beginners. We are providing a detailed manual covering the installation of the tools as well as the complete analysis of a test data set. The test data set is already available inside CoMA and can be analyzed without the need to download it first from a repository. Each step of the workflow is explained in detail and additional literature is provided where it might be useful. Our experience is that especially entry-level users often struggle with solely command-line-based tools. Nevertheless, they can consult the source code any time and learn its’ usage step by step.

***3. It reported that "the workflow results in highly esthetic and publication-ready graphics". However, I didn't find any example figure in the manuscript and CoMA's website. As I didn't run CoMA, so I cannot check how good the output figures are. I think the authors should provide a demo/tutorial or some example output figures on their website.***

DONE. Thank you for this great suggestion! We have included a demo gallery showing the different graphics that can be created with CoMA to our webpage (<https://www.uibk.ac.at/microbiology/services/coma.html>). Certainly, this will help to get a better idea of CoMA before installing it.

***4. It reported that "we used various open-source, third-party tools and combined them into a linear analysis workflow in the form of a Bash script". Hence, I think the core of CoMA is just a bash script. I think the authors need to make their code available (maybe on GitHub) if CoMA is an open source software.***

CoMA is implemented as Bash script calling both, already available open-source tools as well as in-house Python scripts. Particularly the steps for data visualization and statistical appraisal are mainly done using our own scripts. We will provide all code on a public repository (GitHub) upon official publication of the CoMA pipeline. The information on the access to this code is now given in the manuscript; please see line 871.

***5. In addition, the authors didn't describe which tools are used in CoMA and in which steps they are used. In Figure 1, the main tools used in each step need to be marked.***

All third party tools are provided in the “Implementation of CoMA” section (2.1.) of the manuscript. A complete summary is also included in the CoMA manual (chapter “Software list”). However, we did not include this information to Figure 1 since most steps are done by a combination of third party tools and CoMA scripts. Moreover, we wanted to keep the depiction of the workflow as concise as possible.

***6. It reported that the results generated by CoMA and QIIME were very similar. Is it because CoMA used QIIME in several key steps?***

This is not the fact, QIIME scripts are only used in two steps within CoMA, namely removal of rare OTUs and subsampling. Both steps are called after the creation of the OTU table and no major effect on the overall results is expected. Moreover, the removal of rare OTUs and subsampling was also done with the same QIIME scripts on the OTU tables originating from Mothur, QIIME and QIIME2.

***7. For Soil dataset, QIIME2 wasn't included in the comparison. Why?***

For the soil data analysis, we decided to focus on OTU-based approaches since the majority of current soil studies still compares OTUs. Moreover, we wanted to focus clearly on the biological interpretation of the data in this section, and believe that tackling also the issue of OTU versus ASV would be beyond the scope here.

***Minor comments:***

***1. Line 126, delete the space in the URL.***

DONE.

***Reviewer #3***

***The authors present a novel practical pipeline for processing of next-generation datasets for microbiome analyses. The modules in this pipelines range from QC to taxonomic assignment and clustering analysis. The pipeline also provides visualization and statistical analysis tools. The pipeline is bench-marked on multiple microbiome datasets such as soil, mock datasets. The experiments suggest that it outperforms other known methods in the field.***

***The paper is well-written and is a good contribution to the community. A few minor concerns are:***

Thank you very much for your positive feedback, we highly appreciate it! Please find below our individual responses to the points you raised.

***1) A figure representing more details of the tasks (QC, taxonomic assignment) will be helpful.***

DONE. We have added additional information on the taxonomic assignment step to the caption of Figure 1. The quality control steps in CoMA call the PRINSEQ tool, which produces a quality report for each sample. We have decided to leave this out of Figure 1 since it might not be relevant for most readers at this stage. However, we have included now the additional information in the chapter “Data pre-processing and quality checking”; please see lines 157-159.

***2) One feature that is encouraged in the pipeline is to compare and validate across technologies.***

I hope we are interpreting this point raised by the reviewer in the right way. Generally, all tested pipelines (CoMA, Mothur, QIIME and QIIME2) performed well and revealed the general pattern of the mock communities correctly. However, there were minor differences between the individual results, which can be explained by the different approaches/algorithms used by each tool. Which pipeline is performing best highly depends on the analyzed dataset and its structure as well as on the applied user settings. We recommend adjusting the selected pipeline individually based on the characteristics of the dataset and always test different settings. Applying a second tool may help validating the results, particularly when analyzing unfamiliar data.

***3) With upcoming HiFi technology, there is a huge opportunity to perform strain-aware metagenome assembly. The computational methods like SDip (please cite https://doi.org/10.1101/2020.02.25.964445) can be explored for assembling microbiomes. This method has the potential to use variation from multiple strains and species to bin reads and perform assembly. A few sentences on third-generation technologies and analysis methods in the manuscript are appreciated.***

DONE. We are providing some information on third-generation sequencing in the introduction chapter and provide the SDip citation as suggested; please see lines 117-120. However, the focus of this paper is short reads yielded from amplicon sequencing. The processing of long reads and the application of different genome assembling strategies was not covered by our study.
