# Supplementary material for: CoMA – an intuitive and user-friendly pipeline for amplicon-sequencing data analysis
Source: PLoS One. 2020 Dec 2;15(12):e0243241. doi: 10.1371/journal.pone.0243241 (PMC7710066; doi:10.1371/journal.pone.0243241)
Supplement: Attachment — Submitted filename: Response to Reviewers.docx [file pone.0243241.s011.docx]

**PONE-D-20-21940R1**

***Reviewers' comments***

Below are the responses to the Reviewers’ comments. The line numbers we are referring to are those of the revised manuscript (“Revised Manuscript with Track Changes.docx”).

***Reviewer #1:***

***L 70 ITSoneDB is the correct name***

DONE. Changed accordingly.

***L 71-74 Please clarify what did you mean with “de novo assembling”***

DONE. We have revised the sentence in order to sort out any uncertainties. Please see lines 67 – 71.

***L 170-172 Authors stated in COMA a denoising and error correction step is implemented by using Lotus. It is not clear what they mean with “denoising and error correction”. Literally denoising is the procedure applied to remove the noise introduced by amplification and sequencing, as stated by Quince et al in the paper describing AmpliconNoise. Actually, the denoising procedures are strictly related to the used sequencing platform as well as different sequencing approaches are characterized by different error profiles. In the introduction, authors declare COMA is able to analyze Illumina, PacBio and Roche 454 data, consequently if a denoising step is implemented in the pipeline, it must take into account the different peculiarities of these sequencing approaches. Authors should clarify this point.***

***“The quality filtering is done using LotuS, precisely the sdm tool. Regarding the LotuS publication (***[***https://doi.org***](https://doi.org) ***/10.1186/2049-2618-2-30), sdm “... filter(s) input sequences after average quality, accumulated error over the sequence, quality in a freely definable window and remove 5′ low-quality bases filtered for these criteria. Furthermore, sequences are filtered for min/max length, ambiguous nucleotides, max barcode and primer errors, polynucleotide runs, and trimmed for adapter sequences, if present.”. The reviewer is right in the sense that the filtering settings largely depend on the sequencing platform; hence, LotuS provides pre-adjusted sdm setting files for MiSeq, HiSeq, 454 and PacBio. The newest version of the CoMA pipeline now asks the user, which sequencing platform was used, and selects the corresponding sdm file for the sequence filtering process.”***

***Filtering and denoising are two different things. Based on the authors answer they performs filtering and not denoising. Please fix this point in the manuscript.***

DONE. We removed the term “de-noising” in order to avoid any further confusion. Please see lines 166 – 168.

***Moreover, the ASV inference pipeline implemented in QIIME2 is deeply influence by the used filtering parameters. Have the authors used the default parameters or have adjusted them according to the overall data quality? Finally, authors should indicate the versions of the used tools.***

***“DONE. As for QIIME and Mothur, we also used the recommended settings for the QIIME2 analysis from the official SOP documentation. We are providing the applied versions of Mothur, QIIME and QIIME2 now in the manuscript; please see lines 260, 292 and 311.”***

***The QIIME2 SOP documentation the authors mentions are tutorials designed according to data characteristics and consequently may not work correctly on your data.***

DONE. We are sorry for being not sufficiently precise in our response the last time. We followed the QIIME2 SOP in terms of which steps were applied. However, we always considered the sequence quality for determining suitable filtering parameters (e.g. analyzing the output of “qiime demux summarize”). We slightly revised the Material and Methods section describing the QIIME2 data analysis workflow in order to clarify this point; please see lines 318-322.

Addition remark:

Since Reviewer 2 also criticized the length of the manuscript, we have decided to move the parts describing the microbial aspects of the soil samples to the supplementary material (S1 File) as you suggested during the previous round of revision. We believe that this makes the manuscript now better focused on the main purpose of the publication: the presentation and evaluation of the CoMA pipeline. Thank you for this useful suggestion!

***Reviewer #2:***

***The authors' responses and revisions addressed some of my comments in the last round of review. However, the most important comments were not well addressed. Generally, I think the core purpose of the manuscript is to introduce the new pipeline CoMA. So I think the comments focusing on the usability of the pipeline are the most important.***

***1. I suggested using Docker instead of VirtualBox .ova file to distribute the pipeline. I am glad that the authors agreed with my suggestion. They said they will develop "Docker image, a direct Linux installer as well as Singularity container" of CoMA and provide all code on GitHub soon (two or three weeks). However, I think all these things need to be well-prepared and described in the revised manuscript before this submission of revision.***

DONE. The alternative installation options (Singularity image, Linux installer) are available now on the CoMA webpage (<https://www.uibk.ac.at/microbiology/services/coma.html>). We updated the manuscript (please see lines 119-121 and 741-748) as well as the manual (chapters ii and iii) in order to describe all installation options. Moreover, all CoMA source files are now uploaded and publicly available on GitHub (<https://github.com/SebH87/coma>).

***2. It seems that the other two reviewers didn't test the CoMA VM in the last round of review either. Although the CoMA VM would cost a lot of my disk space, I think I should install and test it. However, the usability of CoMA is worse than I expected.***

***2.1. In the beginning, it couldn't work. I checked the log file and found there is a kind of conflict between my MacOS 10.15.6 and VirtualBox (***[***https://forums.virtualbox.org/viewtopic.php
?f=8&t=99739***](https://forums.virtualbox.org/viewtopic.php?f=8&t=99739)***). Then I disabled the mic device in VirtualBox and it worked. Fortunately, it only took 19GB on my Mac instead of the 50GB suggested space.***

We are sorry to hear that you encountered problems when installing CoMA. It seems that there is a problem with audio permissions when using VirtualBox on MacOS. This, however, is a VirtualBox issue and we cannot solve it from our side. Nevertheless, we are describing this problem now in the updated CoMA manual in order to making users aware of the problem; please see chapter i.ii. (Notes). Thank you very much for sharing this important issue!

We are glad to provide now also alternative options for installation (please see point 1) so that users can work with CoMA even in such (rare) cases in which VirtualBox is not working properly.

The 50 GB of disk space are just required during the installation (it is the dimension of the virtual hard drive of CoMA). Thereafter, only disk space you are actually using (+ ca. 20 GB for the Linux system and all required software) will be occupied. If you have severe problems with disc space, you should consider using one of the other installation options (e.g. Singularity).

***2.2. Then I followed the CoMA manual to install usearch and then test it with the example data in CoMA VM*** [***https://www.uibk.ac.at/microbiology/services/manual-coma.pdf***](https://www.uibk.ac.at/microbiology/services/manual-coma.pdf)***. I still don't agree that the GTK dialog is a user-friendly way. In fact, the CoMA GTK dialog doesn't provide more information than directly display and input in the command line terminal.***

For sure, both options have their advantages and drawbacks. As already mentioned, we decided to use the GTK dialogs over direct terminal input based on the feedback of several test candidates, most of them entry-level users.

***It's even worse because I couldn't trace back all the parameters I inputted in the GTK dialog window. The pipeline output all the information to the terminal.***

You can trace all input parameters by checking the log file. Moreover, all parameters are shown in the Terminal window. It could be that you used the old version of CoMA (1.0). This has been improved in the newest version (CoMA 2.0); details where to find this info is given in the newest manual (chapter iv - Tutorial).

***The authors should redirect log information to separated log files of each tool, and keep the terminal only display a few main information.***

Maybe you used the old version of CoMA (1.0)? Actually, CoMA 2.0 only displays the most important outputs (including parameters set by the user) in the Terminal window. This output is also stored in a log file for each run (xxx.log) so that the users can check all settings later on. All other output (warnings, status messages of third party tools, etc.) is not shown in the Terminal and redirected to a second log file (xxx_detailed.log).

***Besides, as I need to test several times with different choices, so I have to go through the whole dialog windows again and again. If it's a command-line tool, I can just write the whole command once and then copy&paste and modify some parameters. Therefore, I don't agree that GTK dialog is a good way even for entry-level users.***

We agree that this is a drawback of our GTK dialog-based approach. Hence, we are planning an advanced mode of CoMA, where the user provides all settings already at the beginning of the run in the future. This might be done using a parameters file that can be handled over to the pipeline. This way, users do not have to type in their parameters continuously. Moreover, this advanced mode will facilitate the use of CoMA on HPC cluster systems, where jobs are typically scheduled.

***2.3. I ran the pipeline strictly following the manual. It showed that I successfully ran the project. Although I saw several "Error" information in the terminal and the final log file, I thought they were just "warnings", such as "Error: (1431.1) FASTA-Reader: Warning: FASTA-Reader: ".***

Thank you for pointing out this issue, this is interesting, as we have never encountered this warning message so far and neither of our several test candidates. Which CoMA version did you use (1.0 or 2.0)? Did you analyze the provided example dataset or your own data? After a check on StackOverflow, it seems as if there could be an issue with BLAST when using too long description lines (> 1000 characters). Maybe this was the case? Truncating the headers might solve the problem. We hope we were able to clarify this issue. We have also added this information in the CoMA manual; please see point 15 of chapter iv (Tutorial).

***2.4. However, I tried some other choices and caused some errors such as Python package biom terminated by "UnboundLocalError: local variable data_start referenced before assignment". The CoMA pipeline didn't show me any useful information about whether it's a software problem or caused by a wrong parameter I chose. The funny thing is that the pipeline still showed "Process succeeded!" even though the error happened. In fact, I found the pipeline totally doesn't do exception handling. It's unacceptable.***

In order to solve this issue, we would need to know at which step you received this error message (and ideally see the log file). Did you use the old version of CoMA? Most likely, the error message comes from a QIIME or Biom-Tools script that might be outdated. When using CoMA 2.0, none of our test candidates encountered such an error. Actually, CoMA does exception handling in various steps in order to prove user input parameters. However, errors originating from third party software (e.g. due to depreciated versions) cannot be caught in most cases. However, for such situations, we are providing a support e-mail service, where users can report their issues and we will fix each of them individually. There is also the plan to include a user forum to the CoMA web page.

***3. I still think it's necessary to revise Figure 1 to show the main tools or parameters in each main step of the pipeline. In fact, I checked the source code of the pipeline in CoMA VM. Besides the python code for plotting and generating reports, there is not much code for the main steps of the pipeline. Therefore, it shouldn't be a problem to illustrate more detail of the main steps of the pipeline in one figure. And it's worth doing.***

DONE. We have modified Figure 1 according to your suggestions and indicate now, where third party tools are used.

***4. In fact, I agree with Reviewer #1's comment that the manuscript is too long and not focused. The authors should describe more details of the pipeline, such as the key parameters used in each step, instead of describing the real dataset.***

DONE. Since two Reviewers criticized this point, we have decided to move the parts describing microbiological aspects of the soil samples to the supplementary material (S1 File). We are confident to better focus on the main purpose of this publication, which clearly is the presentation and evaluation of the CoMA pipeline. Thank you for this critical but important comment!
