# Supplementary material for: CoMA – an intuitive and user-friendly pipeline for amplicon-sequencing data analysis
Source: PLoS One. 2020 Dec 2;15(12):e0243241. doi: 10.1371/journal.pone.0243241 (PMC7710066; doi:10.1371/journal.pone.0243241)
Supplement: Attachment — Submitted filename: Response to Reviewers.docx [file pone.0243241.s012.docx]

**PONE-D-20-21940R2**

***Reviewer’s comments***

We thank Reviewer #2 for his/her further suggestions. Below are the responses to the individual comments.

***Reviewer #2:***

***I am glad that the authors have made a lot changes to their software. I just tested the Singularity version of CoMA v2.0 in this review.***

***Major comment:***

***1. The authors responded some of my comments by asking me which version of COMA was used and telling me that I should use the latest v2.0 for testing. I used v1.0 because there was only v1.0 could be downloaded from their website before this round of review. I don't think I could test the current v2.0 before this round of revision. Now they have updated their website and added v2.0 download links.***

We would like to apologize for this confusion! Following the suggestions of another reviewer, we have provided CoMA 2.0 for download on our webpage several months ago. This download, however, required a user name and password, which we forgot to share with you. Sorry for that! We are glad that you could test the most current version of the tool now by using the Singularity image.

***2. The authors used old Singularity version (v2.x). They should clearly mention it in their manual because current Singularity 3.x is quite different from v2.x. The Singularity commands listed in their manual are unavailable in v3.x, such as "singularity image.create". In fact, I think it's better to use Singularity v3.x to build CoMA v2.0.***

DONE. We are aware of the big differences between Singularity v2.x and v3.x. Our intention was to guarantee most flexibility in usage. However, we agree with you that Singularity v3.x is most relevant nowadays and, hence, have decided to switch completely to this version. Since the creation of overlay images is no longer supported in the 3.x version and needs to be done manually by the users, we have decided to provide an already created overlay image file together with the Singularity image for download.

***3. Singularity is good for HPC. As most of the HPCs are accessed by SSH, so the users need to set X11 forwarding to use the GUI from Singularity on remote HPC. Besides, add "-B ~/.Xauthority" parameter to command "singularity shell". It should be mentioned in their manual. Otherwise, the users might be not able to run the CoMA GUI correctly because it's not common to use GUI from Singularity. Besides, it might be more difficult to set this on Mac and Windows. The manual just say that it can be used on MacOS and Windows, but it didn't give any instructions.***

DONE. Thank you for pointing out this important aspect! We have added a comment to the manual clarifying that X11 forwarding needs to be activated when using a SSH connection. This is indeed crucial since most HPC systems are accessed via SSH.

DONE. We believe that the additional option "-B ~/.Xauthority" is not necessary in most cases since Singularity binds the host system’s Home directory anyway when using the default settings of the tool. Nevertheless, we have added it now in order to guarantee functionality also in case of altered Singularity settings.

You are right; we are not explaining the usage of Singularity on Windows/MacOS in detail. We clearly encourage using VirtualBox for these operating systems, and Singularity (or the direct Linux installer) for Linux/HPC users. However, if anyone is still interested, we are providing links to the instruction page of Singularity (please see point ii.ii of the CoMA manual). The installation is explained here in detail and we do not believe that it is necessary to repeat it in the CoMA manual.

***4. There might be some problems in the step of renaming samples. For the tutorial, it only shows sample 2 and 3. Sample 1 was missing and it caused errors in downstream analysis. If I chose "Don't rename", then I can get results of downstream analysis.***

***Renaming of samples in progress ...***

***Please enter the new name for each sample:***

***2:***

***S2***

***3:***

***S3***

***Process succeeded! Your samples are renamed now!***

Thank you for reporting this. We double-checked the respective Python script and did not find any mistake that might cause such a behavior. In addition, neither of our test candidates reported a similar issue. We assume that your current OTU table file (“otu_table.txt”) might be corrupted for some reason. There is also the possibility that Sample 1 got lost in course of the subsampling process (a sample is discarded if the subsampling depth is higher than its read count). To clarify this, we would need to know how many samples are shown in the OTU-table before the renaming step and how many in the original OTU-table (which is the version directly created by LotuS).

***5. For my previous comment point 2.4, I meant that CoMA cannot correctly check and handle potential error input of files or parameters. For example, if I input only one FASTQ file from the provided example files, and chose "paired end". It's definitely a naive mistake of user's input, but CoMA pipeline doesn't check this kind of mistakes and it continues. The main terminal was stuck at the "Trimming process proceeding ... " step and the size of detail log file increased with a lot of error message. It's just a very simple problem caused by parameter conflicts. I think CoMA should check the conflicts of parameters and give the users opportunity to re-input the parameters. In current CoMA v2.0, even I found that I just input a wrong parameter in previous step, I cannot change it and I have to stop and re-run the pipeline from the first step.***

DONE. We agree that not checking for correctly assigned input files was problematic. Hence, we have decided to implement a step that is checking the user input during sample registration as well as the provided input files. CoMA now warns the user and terminates the run, if the common part of the sequences was not entered correctly as well as if paired-end samples are missing. Moreover, CoMA catches now errors evoked by missing files in multiple situations and prints it in the terminal window. The error messages are short and clear in order to help the user to solve the issue immediately. Thank you for these important inputs; they truly improved the functionality of the pipeline!
